# Bone marrow mesenchymal stem cell-derived exosomes shuttling miR-150-5p alleviates mechanical allodynia in rats by targeting NOTCH2 in microglia

**DOI:** 10.1186/s10020-022-00561-x

**Published:** 2022-11-08

**Authors:** Shuangqing Li, Ciying Huang, Chao Tu, Ruiqi Chen, Xiaolei Ren, Lin Qi, Zhihong Li

**Affiliations:** 1 Department of Anesthesiology, The Second Xiangya Hospital, Central South University, Changsha, 410010 Hunan People’s Republic of China; 2 Department of Ophthalmology, The Second Xiangya Hospital, Central South University, Changsha, 410010 Hunan People’s Republic of China; 3 Department of Orthopedics, The Second Xiangya Hospital, Central South University, No. 139 Renmin Road, Changsha, 410010 Hunan People’s Republic of China; 4 Hunan Key Laboratory of Tumor Models and Individualized Medicine, The Second Xiangya Hospital, Central South University, Changsha, 410010 Hunan People’s Republic of China

**Keywords:** Bone marrow mesenchymal stem cell, Exosome, miR-150-5p, NOTCH receptor 2, Mechanical allodynia, Microglia

## Abstract

**Background:**

This study probes into the function and mechanism of bone marrow mesenchymal stem cell (BMSC)-derived exosomes loaded with miR-150-5p in mechanical allodynia.

**Methods:**

BMSCs were infected with miR-150-5p inhibition lentiviruses to obtain exosomes with low miR-150-5p expression. A L5 spinal nerve ligation (SNL) model was established in rats where exosomes, NOTCH2 overexpression/inhibition plasmids, or microglial cells were intrathecally administered. Hind paw withdrawal threshold (PWT) and paw withdrawal latency (PWL) of rats were measured. TUNEL staining was used to measure the apoptotic rate in rat spinal dorsal horn (SDH), ELISA to evaluate pro-inflammatory factor levels, and RT-qPCR, western blotting, and immunohistochemistry to detect miR-150-5p and NOTCH2 expression. Immunofluorescence was used for localizing exosomes and NOTCH2 and detecting the expression of OX42, a maker for microglia. Dual luciferase reporter and RNA pull down assays were performed to validate the putative binding between miR-150-5p and NOTCH2.

**Results:**

NOTCH2 expressed at a high level and miR-150-5p was downregulated in SDH of SNL rats. Exosomes injected were localized in rat SDH. BMSC-exosomes or NOTCH2 downregulation increased PWT and PWL of SNL rats and reduced apoptosis and inflammation in SDH. In contrast, NOTCH2 overexpression aggravated mechanical allodynia and SDH injury. Moreover, inhibiting miR-150-5p in BMSC-exosomes offset the therapeutic effects of BMSC-exosomes. Microglia activation induced mechanical allodynia in wild rats, while intrathecal injection of microglial cells incubated with BMSC-exosomes showed alleviated mechanical allodynia in SNL rats. NOTCH2 was targeted by miR-150-5p.

**Conclusion:**

BMSC-derived exosomal miR-150-5p alleviates mechanical allodynia by targeting NOTCH2 in microglial cells.

## Introduction

Neuropathic pain represents the pain caused by an injury to the somatosensory system and affects about 7 to 8% of the general population (Bouhassira 2019). Allodynia and hyperalgesia are two prominent manifestations of neuropathic pain, both of which are seen in a variety of peripheral neuropathies and central pain disorders, affecting 15–50% of patients with neuropathic pain (Jensen and Finnerup 2014). Mechanical allodynia refers to the pain induced by innocuous stimuli that do not usually provoke pain. A wide range of central mechanisms are involved in the initiation of allodynia, including phenotypic changes in neurons and immune cell-mediated changes (Lolignier et al. 2015).

Microglial cells are professional phagocytes of the brain, which can migrate to different regions of the central nervous system and recognize cells that undergo programmed cell death (Wolf et al. 2017). As the first line of defense, microglial cells are activated in response to pathogen invasion or neuronal debris and release inflammatory cytokines to mediate inflammatory reaction in various neurodegenerative diseases, such as Alzheimer’s disease, Parkinson’s disease, and multiple sclerosis (Xu et al. 2016). Microglial cells are dramatically activated in spinal dorsal horn (SDH) after peripheral nerve injury, which is commonly observed in various models of neuropathic pain (Tsuda 2016). NOTCH pathway is a highly conserved signaling cascade that involves microglia activation in neuropathic pain (Jin et al. 2021). NOTCH receptor 2 (NOTCH2) was found to be a downstream gene of miR-151a-3p, which contributed to neuropathic pain behaviors (Zhang et al. 2021), yet the implication of NOTCH2 in microglia-related neuropathic pain has been rarely mentioned.

Stem cells have shown great therapeutic effects in various neurodegenerative diseases by several mechanisms, including cell replacement/repair, immunomodulation, and stimulation of progenitor cell differentiation (Genc et al. 2019; Kang et al. 2016; Venkatesh and Sen 2017). Also, stem cells can produce extracellular vesicles including exosomes which deliver mRNAs, microRNAs (miRNAs), and proteins to recipient cells or tissues to induce nonautonomous changes that are therapeutical (Phinney and Pittenger 2017). Stem cell-derived exosomes show a great potential as cell-free regenerative medicines. A study has reported that exosomes derived from human umbilical cord mesenchymal stem cells (MSCs) reduce neuropathic pain by acting on neurons and glial cells (Shiue et al. 2019). However, the molecular mechanism for exosome-mediated mechanical allodynia remains undefined to date.

MiRNAs are a major class of small noncoding RNAs which can be guided to the 3’ end of their target mRNAs through base pairing and result in destabilization and translational repression of the mRNAs (Saliminejad et al. 2019). Dysregulation of miRNAs is implicated in a wide range of human diseases. Several miRNAs have been reported for their involvement in neuropathic pain. For instance, miRNA-23a reduced neuropathic pain by inhibiting TXNIP/NLRP3 inflammasome axis via CXCR4 in spinal glial cells (Pan et al. 2018). In contrast, miRNA-30c-5p expression was upregulated by chronic peripheral ischemia and exhibited a predictive value for the severity of allodynia (Tramullas et al. 2018).

This research established a rat model of neuropathic pain to study the therapeutic effect of exosomes derived from bone marrow mesenchymal stem cells (BMSCs). Bioinformatics analysis showed that NOTCH2 had several miRNA-binding sites. Among these miRNAs, miRNA-150-5p (miR-150-5p) is abundantly expressed in BMSC-derived exosomes (BMSC-exosomes). Therefore, we hypothesized that BMSC-exosomes might act on mechanical allodynia by delivering miR-150-5p to microglial cells to suppress the expression of NOTCH2. Animal experiments were conducted to validate this hypothesis in this study.

## Materials and methods

### Experiment animals

Five male Sprague–Dawley (SD) rats (four weeks old, 80–100 g) for BMSC extraction and 102 healthy male SD rats (eight weeks old) for spinal nerve ligation (SNL) were acquired from Huafukang Biotechnology Co., Ltd. (Beijing, China). Rats were reared in specific pathogen-free rooms at constant room temperature (21–25 °C) and humidity (50–65%) with 12-h light–dark cycles and free access to food and water. All animal experiments abided by the rules and regulations of laboratory animals. The experimentation in the study was ratified by the ethics committee of the Second Xiangya Hospital.

### Isolation of primary rat BMSCs

Five four-week-old male SD rats were anesthetized with 2% pentobarbital sodium (50 mg/kg) by intraperitoneal (i.p.) injection. The bilateral femurs and tibiae of the rats were collected and the bone marrow cavity was exposed and washed repeatedly with α-MEM (Gibco, Grand Island, NY, USA) until the bone turned pale. Muscle tissue debris in the fluid was removed with a bacteria-proof filter, and the cell filtrate was centrifuged at 2000 rpm for 10 min. The supernatant was discarded and the cell pellet was resuspended in α-MEM supplemented with 10% fetal bovine serum (FBS). The cells (1 × 10^6^) were then cultured at 37 °C with 5% CO_2_ and saturated humidity. After cell culture for 3 days, the cells were observed and the medium was replaced and refreshed every 2 ~ 3 days thereafter. Upon reaching 80 ~ 90% confluency, cell passage was carried out at a ratio of 1:3.

### Identification of BMSCs

BMSC culture medium was refreshed every three days and the BMSCs were obtained by several times of digestion. The BMSCs at a confluency of 80% were passaged and the third generation (P3) of the BMSCs was obtained to observe their morphology and growth status. The proliferative ability of BMSCs was tested using the MTS method, and flow cytometry detected the expression of stem cell markers (positive markers: CD29, CD44, CD73, CD90, and CD105; negative markers: CD34, and CD45).

BMSCs underwent Alizarin red staining (Solarbio, Beijing, China), Oil red O staining (ab150678, Abcam, Cambridge, MA, USA), and Alcian blue staining (BP-DL241, Nanjing SenBeiJia Biological Technology Co., Ltd., Jiangsu, China) to evaluate their capabilities to differentiate into osteoblasts, adipocytes, and chondrocytes. After identification, only the P3 BMSCs were used in subsequent experiments.

### Alizarin red staining

Alizarin red dye was used to detect the osteogenic differentiation potential of BMSCs strictly according to the manufacturer’s instructions. Briefly, after osteogenic differentiation induction for 14 days, osteogenic induction medium was removed and BMSCs were washed twice or thrice in PBS, fixed for 15 min at room temperature, washed with ddH_2_O, and stained with alizarin red at room temperature for 30 min, after which the BMSCs were rinsed in ddH_2_O and observed under an IX50 microscope (Olympus, Japan).

### Alcian blue staining

After 14 days of chondrogenic differentiation induction, BMSCs were washed twice in phosphate buffer saline (PBS), fixed in 4% paraformaldehyde (PFA) (Sigma-Aldrich, St. Louis, MO, USA) for 30 min, and washed thrice with PBS. Afterwards, the BMSCs were dyed with Alcian blue solution for 30 min, followed by washing thrice with PBS and microscopic observation (IX50, Olympus, Japan).

### Oil red O staining

Fourteen days after adipogenic differentiation induction, BMSCs were fixed in 4% PFA for 10 min, washed thrice with distilled water, and stained with Oil red O solution for 30 min. Afterwards, 60% isopropanol was used to remove excess Oil red O solution in culture plates and the cells were gently washed with distilled water until the cleaning solution became clear, and then images were captured with an IX50 microscope (Olympus, Japan).

### Isolation and identification of BMSC-exosomes

BMSC-exosomes were extracted according to the method reported by Yan et al. (2018). Briefly, BMSCs (1 × 10^5^) were plated in six-well plates containing MSC growth medium. After 24 h of cell cultures, the BMSCs were washed thrice with PBS and future cultured for 48 h in exosome-free FBS-contained α-MEM medium (iCell-0650, iCell Bioscience Inc., Shanghai, China). Then the culture (2 mL) was collected and centrifuged at 500 g and 4 °C for 15 min to remove cells, at 2000 g for 15 min to remove cell debris or apoptotic bodies, and at 10,000 g for 20 min to remove large extracellular vesicles. After centrifugation, the supernatant was obtained and filtered with a 0.22 μm filter membrane. The obtained supernatant was centrifuged at 110,000 g for 70 min to avoid protein contamination, and resuspended in 1 × PBS and stored at − 80 °C.

Isolated exosomes were identified using particle size analysis with a NanoSight NS300 instrument (Malvern, UK), transmission electron microscopy, and western blots for exosome marker proteins. In brief, 10–20 μL exosomes were diluted to 1 mL with PBS and analyzed using a NanoSight NS300 instrument at 25 °C and a constant flow rate for particle size analysis; 20 μL exosomes were added onto a copper grid, placed at room temperature for 2 min, negatively stained with 3% phosphotungstic acid solution (12501-23-4, Sigma-Aldrich), washed thrice in PBS, and observed with a Hitachi H7650 transmission electron microscope (Tokyo, Japan); the expression of exosome markers (CD81, CD63, and CD9) and an endoplasmic reticulum marker Calnexin was detected by western blotting (see in western blot part for article numbers of antibodies and detail methods).

### Intrathecal catheterization and administration

Intrathecal catheterization was done reference to the methods described by Shih et al. (2012). After anesthesia with i.p. injection of 2% pentobarbital sodium (50 mg/kg), rats were in prone positions, their lumbar region was raised, and their limbs were fixed. Following disinfection, a 1-cm longitudinal incision was made at the L4-5 vertebrae on the back of the rats. The muscles and fascia were bluntly separated, and a 22-gauge puncture needle, with its bevel cephalad and upward, was inserted into the rat spinal dura mater at an angle of approximately 20° to the rat spinal cord. Tail-flicking indicates that the puncture needle is placed in the subarachnoid space. A PE-10 catheter was placed into the needle and gently advanced 3 cm rostrally at the level of the spinal cord lumbar enlargement segments. At this time, there was cerebrospinal fluid (CSF) flowing in the catheter and CSF leakage was observed at the puncture site. The catheter was secured to the fascia and exited from the back of the neck. About 3 ~ 4 cm of the catheter was left outside, fixed, and washed with saline using a microsyringe. The external end of the catheter was heated and blocked to avoid CSF leakage. Finally, the muscle and skin layers were sutured. All the procedures were operated under sterile conditions and penicillin-G sodium (40,000 U/kg) was given post operation by i.p. injection to avoid infection. Twenty-four hours after recovery from anesthesia, 20 μl of 2% lidocaine solution was injected into rats through the catheter. The catheterization was deemed successful if the rats had temporary paralysis of both lower limbs within 30 s and recovered in few minutes.

### SNL of the fifth lumbar spinal nerve (L5) in rats

A SNL rat model was established reference to the methods described in a previous study (Jaggi et al. 2011). Rats were anesthetized by 2% pentobarbital sodium (50 mg/kg, i.p. injection) and placed in prone positions. A total of 10 cm^2^ fur in the back was shaven off along the intercrestal line. After disinfection, a 1.5-cm longitudinal incision was made 0.5 cm to the left side of the intercrestal line. The skin, fascia, and muscles were bluntly dissected to expose the angle between L5 transverse process and sacrum. The L5 transverse process was wiped with a sterile cotton ball and cut off by elbow hemostatic forceps to expose L4-5 spinal nerves. The L5 spinal nerve was separated and ligated, during which the nerve should be prevented from pulling and was ligated twice in case the thread was detached. Sham-operated rats only underwent L5 spinal nerve exposure and other procedures were performed as in SNL rats. The whole operation was completed under sterile conditions and penicillin-G sodium (40,000 U/kg) was administrated (i.p.) post operation to prevent infection.

**Scheme 1:**
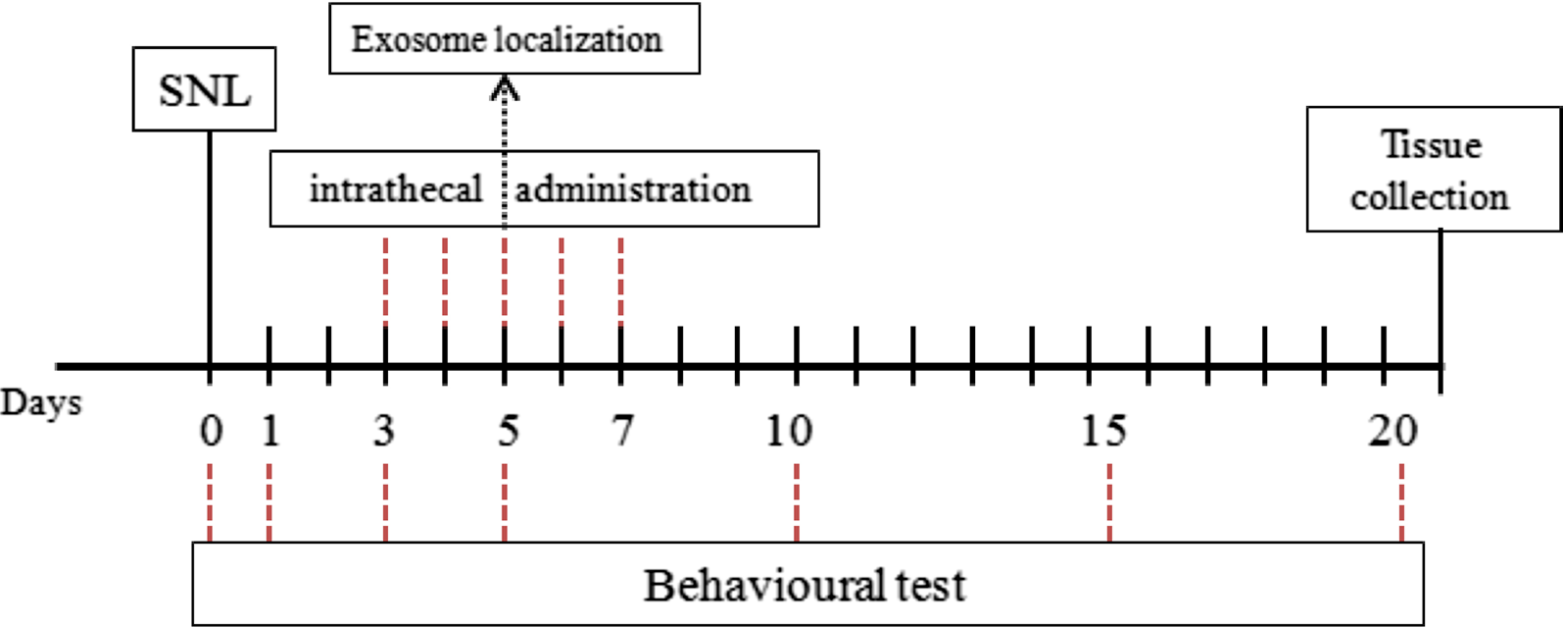
In vivo experimental design

Intrathecal administration of 1 mg/ml exosomes (10 μl), 1 mg/kg NOTCH2 overexpression plasmids (pcDNA3.1-NOTCH2), negative control of pcDNA3.1-NOTCH2 (pcDNA3.1), 1 mg/kg NOTCH2 interference plasmids (sh-NOTCH2), or negative control of sh-NOTCH2 (sh-NC) was performed on SD rats from days 3–7 after operation (See in Scheme 1 for in vivo experiment design). There were 24 rats in the sham group and 78 rats in the SNL group. The above-mentioned plasmids were provided by Shanghai Genechem Co., Ltd. (Shanghai, China).

The sham-operated rats (n = 24) were divided into the sham (n = 12), PBS (sham-operated rats intrathecally injected with ATP-stimulated 1 × 10^3^ cells/10 μL microglial cells that were not pretreated with exosomes, n = 6), and BMSCs-exo (sham-operated rats intrathecally injected with 1 × 10^3^ cells/10 μL microglial cells pretreated with BMSCs-exosomes and stimulated with ATP, n = 6) groups. Behavioral tests were carried out one day before and 1, 3, 8, and 13 days after intrathecal administration.

Seventy-eight rats in the SNL group were divided into the SNL (n = 18), exo (rats subjected to SNL and injected with BMSCs-exosome, n = 18; exosome localization was carried out in six of them), sh-NC (rats subjected to SNL and injected with sh-NC, n = 6), sh-NOTCH2 (rats subjected to SNL and injected with sh-NOTCH2, n = 6), pcDNA3.1-NC (rats subjected to SNL and injected with pcDNA3.1, n = 6), pcDNA3.1-NOTCH2 (rats subjected to SNL and injected with pcDNA-NOTCH2, n = 6), Exo + pcDNA3.1-NOTCH2 (rats subjected to SNL and injected with BMSCs-exosome and pcDNA3.1-NOTCH2, n = 6), LV-anti-miR-150-5p (SNL rats injected with exosomes derived from BMSCs transfected with miR-150-5p inhibition lentiviral vectors, n = 6), and vector-BMSCs (SNL rats injected with exosomes derived from BMSCs transfected with the negative control of the miR-150-5p inhibition lentivirus, n = 6) groups. The doses of the recombinant lentiviruses were 8 × 10^3^ transduction units (Zhang, Gao, Li, Wen, Yan, Peng and Xiao 2021).

### miR-150-5p inhibition in exosomes

BMSC-exosomes expressing miR-150-5p at a low level were obtained by transfection with miR-150-5p inhibition lentivirus (LV-anti-miR-150-5p, 50 nM) or negative control inhibition lentivirus (vector) into BMSCs, and exosomes were extracted 48 h after transfection. Lentiviruses were acquired from Shanghai Genechem Co., Ltd. (Shanghai, China).

### Behavioral testing

Paw withdrawal threshold (PWT) was assessed according to the method developed by Chaplan and colleagues (Chaplan et al. 1994). Specifically, in a quiet environment, rats were placed in a Perspex cage with a mental-mesh floor for 30–60 min of acclimatization, and PWT was evaluated with a von Frey 2390 mechanical pain detector after the rats calmed down. The von Frey filament was used to press the plantar surface of hind paws vertically. The stimuli were lasted ≤ 4 s. When the rats presented paw licking, lifting, or latency, the pressure value on the screen was recorded as PWT. Each rat underwent von Frey tests thrice with intervals of 5 min, and the obtained data were averaged.

Paw withdrawal latency (PWL) was measured reference to the methods reported in a previous study (Chen et al. 2015). In a quiet environment, rats were placed in a Perspex cage with a 3 mm-thick glass floor, with acclimatization to the environment for 30–60 min. After the rats calmed down, the plantar surface of the hind paws was stimulated with infrared radiant heat. When the rats had paw licking, lifting, or latency, the timer was stopped and the time (PWL) was automatically recorded. The cutoff time was 20 s to prevent tissue damage. Each rat was tested thrice with intervals of 5 min and the results of three independent tests were averaged.

### ELISA

Expression levels of TNF-α, IL-1β, and IL-6 were measured using TNF-α (PRTA00), IL-1β (PRLB00), and IL-6 (PR6000B) ELISA kits (R&D Systems, Minneapolis, Minnesota, USA) strictly following the instructions. First, 100 μl samples or standards were added into plate wells and incubated at 37 °C for 90 min, followed by incubation with specific antibodies and avidin–biotin-peroxidase complex solution for 60 and 30 min, respectively. After 20–25 min of color development with TMB, absorbance values at 450 nm were evaluated using a microplate reader.

### TUNEL staining

Rat SDH tissues were fixed overnight in 4% PFA, embedded in paraffin, and cut into paraffin sections. After deparaffinization and hydration, the tissue sections were immersed in 0.25% Triton X-100 at room temperature for 20 min, and fresh TUNEL reaction mixture was added to the tissue sections and incubated at 37 °C for 1 h in the dark. The tissue sections were sealed with anti-fade reagent containing DAPI. After sealing, the sections were visualized under a fluorescence microscope and the apoptotic rate was analyzed using Image J software [apoptotic rate (%) = number of TUNEL positive cells/number of total cells × 100].

### Immunofluorescence

Exosomes were labeled green by an Exo-Glow labeling kit (System Bioscience Inc., Palo Alto, CA, USA) before intrathecal administration. On days 5 post operation (days 3 after exosome injection), six rats were randomly selected and euthanatized to collect their L5 SDH tissues for immunofluorescence.

Rat L5 SDH tissues were collected and cut into paraffin sections after dehydration and embedding. Following deparaffinization, the sections were immersed in TBS and boiled in a microwave oven for 10 min of antigen repair. After sealing at 37 °C for 30 min in bovine serum albumin, the sections were incubated with diluted primary antibodies of NOTCH2 (ab118824, 1:100, Abcam) and OX42 (GTX76060, 1:50, GeneTex, USA) at 4 °C overnight. The sections were then incubated with a secondary antibody for 1 h, washed with PBS, sealed with DAPI, and observed under a fluorescence microscope.

### Immunohistochemistry (IHC)

Rat L5 SDH tissues were fixed in 4% PFA for 48 h, cut into 5-μm-thick sections, and roasted for 20 min, followed by deparaffinization in conventional xylene. After washing once in distilled water and thrice in PBS, the tissue sections were evenly covered with 3% H_2_O_2_ for 10 min at room temperature and washed thrice with PBS. After that, the sections were added with goat serum blocking solution and placed at room temperature for 20 min. The sections were incubated with NOTCH2 antibody (ab118824, 1:100, Abcam) at 4 °C overnight. After being washed thrice with PBS, the sections were incubated with a secondary antibody (ab6728, 1:1000, Abcam) at room temperature for 1 h, followed by another round of washing. The sections underwent color development with DAB for 1–3 min and the nuclei were stained in hematoxylin solution for 3 min before dehydration, transparentization, and sealing. The sections were observed under a microscope (× 200) and three random fields were selected and the images were processed with Image J software for IHC scoring. The semi-quantitative results of the micrographs were evaluated by two experienced pathologists using the double-blind method, and the percentage of positive cells and staining intensity were scored. The score scale for percentage of positive cells was: 0, < 5%; 1, 5–25%; 2, 26–50%; 3, 51–75%; 4, 76–100%, and that for staining intensity was: 0, no staining; 1, light yellow; 2, brownish yellow; 3, tan. The products of the two scores were defined as positive grades: 0, negative; 1–4, weakly-positive; 5–8, positive; 9–12, strongly-positive.

### Isolation and culture of microglial cells

MicrogliaL cells were obtained from SD rats (1–3 days old) reference to the method described in a previous study (Fung et al. 2015). Briefly, rat cortex was separated, the meninges was removed, and the cortex was sliced and digested with trypsin for 15 min; cell suspension was cultured and shaken on an orbital shaker for 4–6 h at 37 °C and 200 r/min, after which the culture medium was collected. Since microglial cells did not adhere firmly to the wall, most of the cells in the collected culture medium were microglial cells. The cells were centrifuged at 1000 r/min for 3 min, the supernatant was removed, and the cells were resuspended in complete medium. The collected cells were inoculated into a new culture flask and named as primary microglial cells (P0). Microglial cells were incubated with BMSC-exosomes or PBS at 37 °C for 24 h before the following experiments.

### Cell transfection

MicrogliaL cells were transduced with miR-150-5p inhibitor/mimic or their respective negative controls (inhibitor/mimic NC) (transfection dose of 50 nM, all from GenePharma, Shanghai, China). Following experiments were conducted 48 h after transfection.

### RT-qPCR

Total RNA was extracted from tissues and cells with the use of TRIzol reagent (Takara, Dalian, China), followed by measurement of RNA concentration and purity using a NanoDrop spectrophotometer. RNA was reverse-transcribed into cDNA with the kit (TaKaRa, Tokyo, Japan). RT-qPCR was performed on a Biosystems 7300 real time PCR system (ABI, Foster City, CA, USA) according to the instructions of a SYBR GreenMix kit (TaKaRa). Each PCR experiment was performed in triplicate, and a PCR system was added with 10 ng cDNA. Gene expression was analyzed by the 2^−ΔΔCt^ method (Soejima and Koda 2008) [ΔΔCt = (Ct _target gene_ − Ct _housekeeping gene_) _experimental group_ − (Ct _target gene_ − Ct _housekeeping gene_) _control group_]. GAPDH and U6 were used as housekeeping genes for NOTCH2 and miR-150-5p, respectively. Each experiment was repeated thrice. Primer design and PCR experiments were carried out by RiboBio (Guangzhou, China) (see in Table 1 for primer sequences).

**Table 1:** Primer sequences

Name of primer	Sequences
miR-150-5p-F	TCTCCCAACCCTTGTA
miR-150-5p-R	GAATACCTCGGACCCTGC-
U6-F	AAAGCAAATCATCGGACGACC
U6-R	GTACAACACATTGTTTCCTCGGA
NOTCH2-F	ATTGTCAAACGGTGTTGGCG
NOTCH2-R	CCCACAGGGCATAAGCAAGA
*GAPDH-*F	CCGCATCTTCTTGTGCAGTG
*GAPDH-*R	ACCAGCTTCCCATTCTCAGC

*F* forward, *R* reverse

### Western blotting

After the cells or tissues were treated with RIPA lysis buffer on ice for 15 min, the lysate was centrifuged for 5 min at 13,000 g, and the concentration of isolated total protein was measured using a BCA kit. The lysate was added with loading buffer and boiled in a water bath for 10 min of denaturation. The loading volume of protein samples was calculated based on the loading quantity (30 μg protein per well). The protein was loaded and separated by electrophoresis (80 V for 3 min and 120 V for 90 min), and transferred onto a 0.22-μm PVDF membrane at 250 mA for 100 min. The membrane was washed thrice (two minutes per time), immersed in 5% skim milk at room temperature for 1 h, and incubated with antibodies of NOTCH2 (ab118824, 1:100, Abcam), CD81 (ab79559, 1:1000, Abcam), CD63 (ab134045, 1:1000, Abcam), CD9 (ab223052, 1:1000, Abcam), Calnexin (ab22595, 1:1000, Abcam), OX42 (GTX76060, 1:50, GeneTex), and GAPDH (ab181602, 1:1000, Abcam) at 4 °C overnight. The membrane was then washed thrice with TBST for 10 min each. Secondary antibodies (ab6721, ab6728, Abcam) were incubated with the membrane at room temperature for 1 h, followed by membrane washing. Enhanced chemiluminescence reagent was used for color development and Image J software for calculating relative protein expression. The results represented the average of three independent experiments.

### Dual-luciferase reporter assay

A binding site between miR-150-5p and NOTCH2 was searched by bioinformatics prediction, and wild sequence of the binding site between miR-150-5p and NOTCH2 or mutated sequence were synthesized and inserted into pGL3-Basic vectors (NOTCH2-wt and NOTCH2-mut). The vectors identified by sequencing were transfected with mimic NC or miR-150-5p mimic into microglial cells. The cells were transfected for 48 h and then lysed. A luciferase assay kit (K801-200, Biovision) and a luciferase reporter gene analysis system (Promega, Madison, WI, USA) were used to calculate luciferase activity, using Renilla luciferase as the internal control. The ratio of Firefly luciferase RLU to Renilla luciferase RLU represented the activation degree of the target reporter gene. Each experiment was repeated thrice.

### RNA pull-down

Biotinylated miR-150-5p probe (miR-150-5p probe) and negative control probe (NC probe) were transfected into microglial cells which were collected after transfection for 48 h, lysed, and incubated with streptavidin-conjugated magnetic beads according to the instructions of a Pierce™ Magnetic RNA–Protein Pull-Down kit (Millipore, Billerica, MA, USA). Briefly, the beads were washed and incubated with the cell lysate at room temperature in a rotator. RT-qPCR was used to detect NOTCH mRNA expression in the eluted complexes.

### Statistical analysis

Data were processed by GraphPad Prism 7 and presented as mean ± standard deviation. *T* test was used for comparisons between two groups, and one-way analysis of variance was used for multigroup comparisons with Tukey’s multiple comparisons test for post hoc multiple comparison. *P* < 0.05 was considered statistically significant.

## Results

### Identification of BMSCs and BMSC-exosomes

**Fig. 1:**
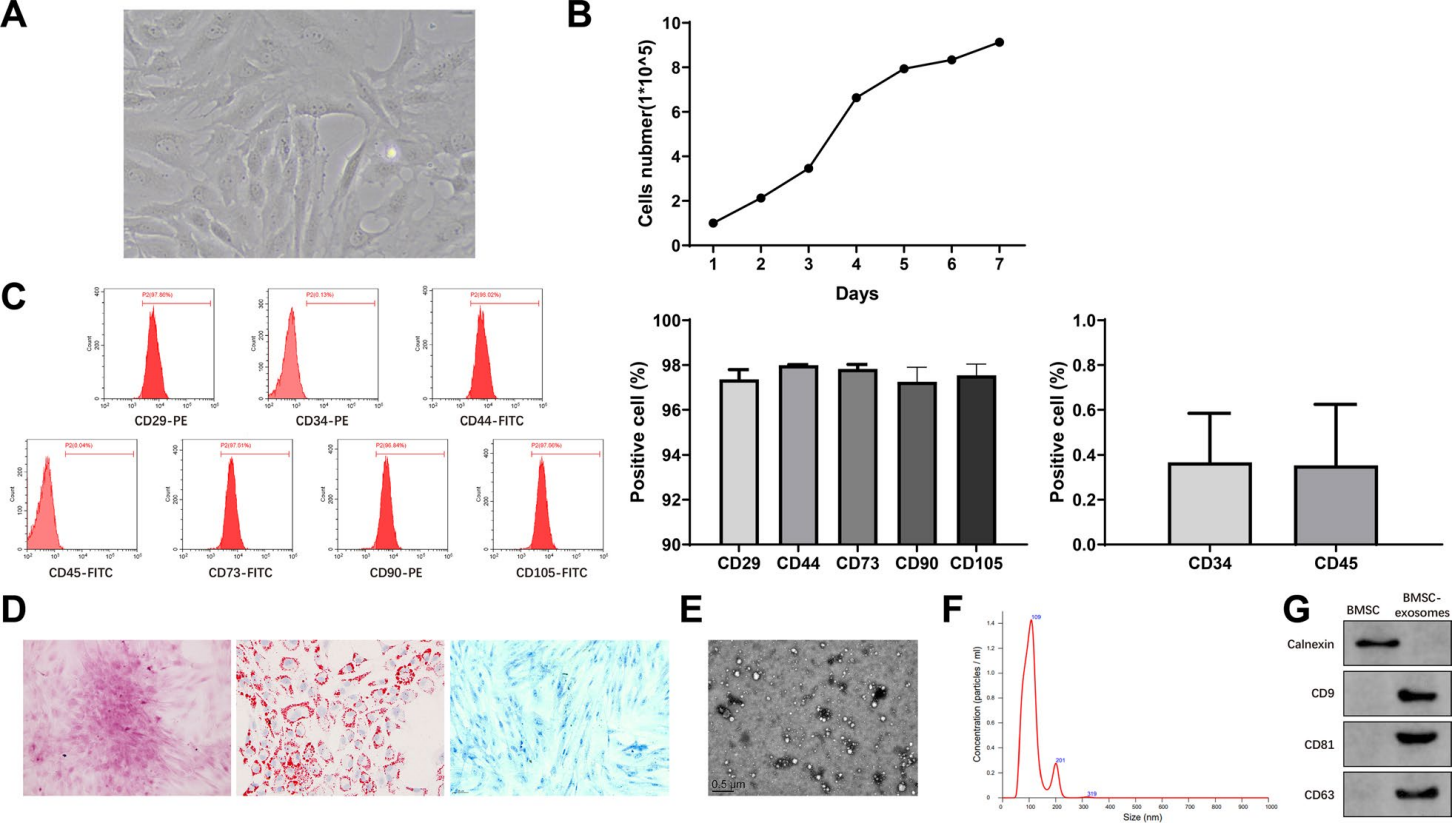
Identification of BMSCs and BMSC-exosomes. **A** Morphology of the third-generation BMSCs; **B** Growth curve of the third-generation BMSCs; **C** Flow cytometry measured the expression of surface markers (CD90, CD73, CD29, CD105, CD44, CD34, and CD45) in BMSCs; **D** Alizarin Red staining assessed the osteogenic differentiation of BMSCs, Oil red O evaluated the adipogenic differentiation of BMSCs, and Alcian blue staining revealed the chondrogenic differentiation of BMSCs; **E** The morphology of the exosomes extracted from BMSCs was observed by transmission electron microscope; **F** Nanoparticle tracking analysis of BMSC-exosomes; **G** Western blotting measured the expression of CD81, CD63, CD9, and Calnexin in BMSC-exosomes. BMSC, bone marrow mesenchymal stem cell

MSCs obtained from bone marrow were observed under a microscope. The P3 cells mainly had spindle shapes, polar growth, and stable morphology (Fig. 1A). The growth curve of the P3 cells was in an S shape, and they grew actively (Fig. 1B). The expression of surface antigen markers for BMSCs in these cells was measured by flow cytometry. The positive rates of MSC markers (CD90, CD73, CD29, CD105, and CD44) were over 95%, while CD34 and CD45 were negatively expressed in these cells (Fig. 1C). Additionally, the results of tri-lineage differentiation indicated that the cells cultured in osteogenic, adipogenic, and chondrogenic differentiation media were positively stained by Alizarin red, Oil red O, and Alcian blue solutions (Fig. 1D). From the above, the isolated BMSCs could be used for subsequent experiments.

Then we extracted exosomes from BMSCs. Under a transmission electron microscope, the exosomes were saucer-like vesicles with double-layer membranes (Fig. 1E). The diameters of the vesicles were 80–160 nm in size (Fig. 1F). Western blotting showed that these vesicles expressed exosome markers (CD81, CD63, and CD9) and did not express the endoplasmic reticulum marker Calnexin (Fig. 1G). These results showed that the vesicles extracted from BMSCs were exosomes without organelle impurities after cell fragmentation.

### BMSC-exosomes reduce mechanical allodynia of SNL rats

**Fig. 2:**
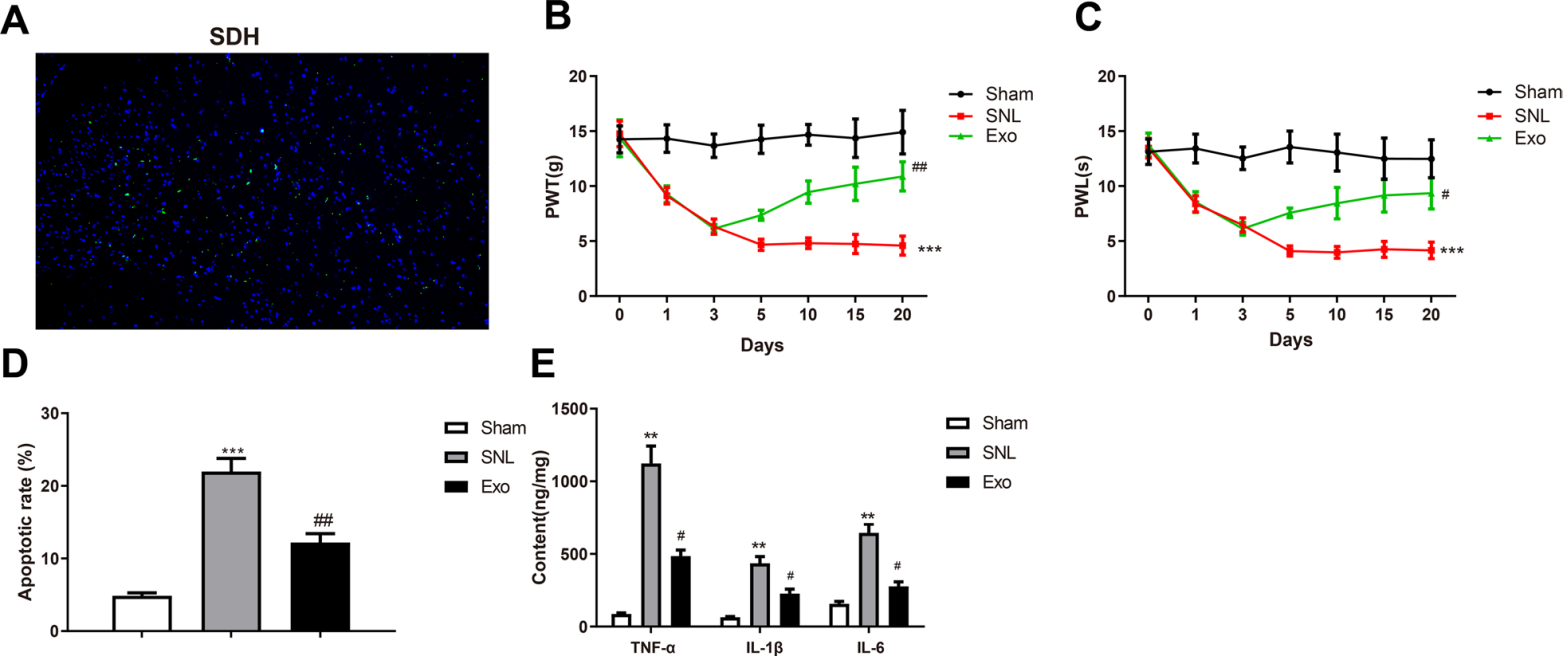
BMSC-exosomes reduce mechanical allodynia of L5 SNL rats. **A** Localization of BMSC-exosomes by fluorescence staining; Hind paw PWT (**B**) and PWL (**C**) of SNL rats on days 0, 1, 3, 5, 10, 15, and 20 after surgery; (**D**) TUNEL staining assessed the apoptosis in rat SDH; (**E**) ELISA measured the expression of TNF-α, IL-1β, and IL-6 in rat SDH. Data were shown as mean ± standard deviation. N = 6. *P < 0.05, vs. the sham group; ^#^*P* < 0.05, vs. the SNL group. *BMSC* bone marrow mesenchymal stem cell, *SNL* spinal nerve ligation, *PWT* paw withdrawal threshold, *PWL* paw withdrawal latency, *SDH* spinal dorsal horn

BMSC-exosomes were intrathecally administrated into rats to evaluate the impact of BMSC-exosomes in neuropathic pain. First, green fluorescence-labeled exosomes were localized in rat SDH, and the result indicated that exosomes transferred to the parenchyma of the nervous system to exert their functions (Fig. 2A). PWT and PWL of rat hind paws were detected to assess the changes in mechanical allodynia and thermal hyperalgesia. Compared with the sham group, the SNL group showed lower PWT and PWL (*P < 0.05); rats in the Exo group had higher PWT and PWL compared with those in the SNL group (^*#*^*P* < 0.01) (Fig. 2B, C), which indicated that exosomes alleviated mechanical allodynia and thermal hyperalgesia in rats.

Apoptosis in rat SDH was evaluated by TUNEL staining. The results showed that the apoptotic rate was significantly increased in the SNL group than in the sham group (*P < 0.05), while that was decreased in the Exo group relative to the SNL group (^*#*^*P* < 0.01) (Fig. 2D). ELISA detected the expression levels of inflammatory cytokines in rat SDH. The expression levels of TNF-α, IL-1β, and IL-6 were elevated in the SNL group compared with those in the sham group (*P < 0.05); those were decreased in the Exo group than in the SNL group (^*#*^*P* < 0.01) (Fig. 2E).

The above results suggested that BMSC-exosomes can improve mechanical allodynia in SNL rats and reduce neuropathic pain-induced inflammation.

### BMSC-exosomes reduce mechanical allodynia via microglia activation

**Fig. 3:**
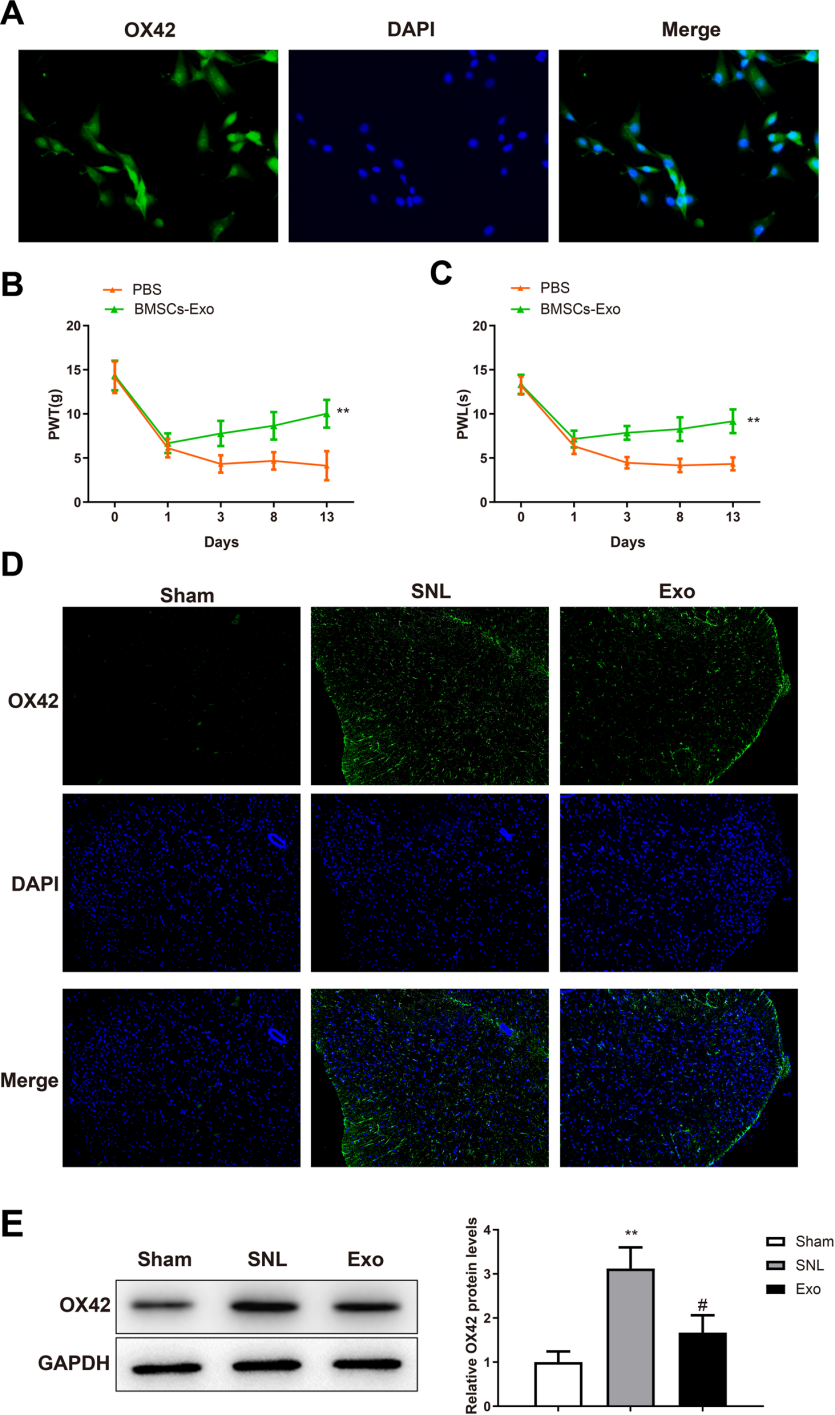
BMSC-exosomes reduce mechanical allodynia by blocking activation of microglia cells. **A** Immunofluorescence was used for microglia identification; hind paw PWT (**B**) and PWL (**C**) of the rats that were intrathecally injected with microglial cells; Immunofluorescence (**D**) and western blotting (**E**) were used to detect the expression of OX42 in SDH. Data were shown as mean ± standard deviation. N = 6. *P < 0.05, vs. the sham or PBS group; ^#^*P* < 0.05, vs. the SNL group. *BMSC* bone marrow mesenchymal stem cell, *PWT* paw withdrawal threshold, *PWL* paw withdrawal latency, *SDH* spinal dorsal horn

Injection of ATP-stimulated microglial cells into the spinal cord has been reported to induce pain hypersensitivity in naive rats and BMSCs can reduce neuropathic pain by inhibiting microglial cell activation (Ferrini et al. 2013; Huang et al. 2018). First, microglial cells were isolated from the SD rat. Western blotting showed that the isolated cells strongly expressed OX42, a specific marker for microglial cells (Fig. 3A). Then, microglial cells were pre-treated with BMSC-exosomes or PBS and stimulated with ATP before intrathecal injection (1 × 10^3^ cells/10 μl) into sham-operated rats. Mechanical allodynia and thermal hyperalgesia were evaluated by detecting PWT and PWL. Compared with those in the PBS group, PWL and PWT were increased in the BMSC-Exo group (Fig. 3B, C, *P < 0.05). The above results indicated BMSC-exosomes alleviated the mechanical allodynia induced by microglial cells and that the ameliorating effect of BMSC-exosomes in mechanical allodynia was involved with microglia cells.

To further investigate the effect of BMSC-exosomes on microglial cells, we detected the expression of OX42 in SDH of SNL rats after intrathecal administration of exosomes. Immunofluorescence showed that OX42 level was significantly increased in the SNL group compared with that in the sham group (*P < 0.05); OX42 expression was substantially downregulated in the Exo group compared with that in the SNL group (^*#*^*P* < 0.01) (Fig. 3D). The immunoblots of OX42 recapitulated the results of immunofluorescence (Fig. 3E). Overall, BMSC-exosomes could inhibit microglial activation in SDH to alleviate mechanical allodynia.

### BMSC-exosomes downregulate NOTCH2 in microglial cells

**Fig. 4:**
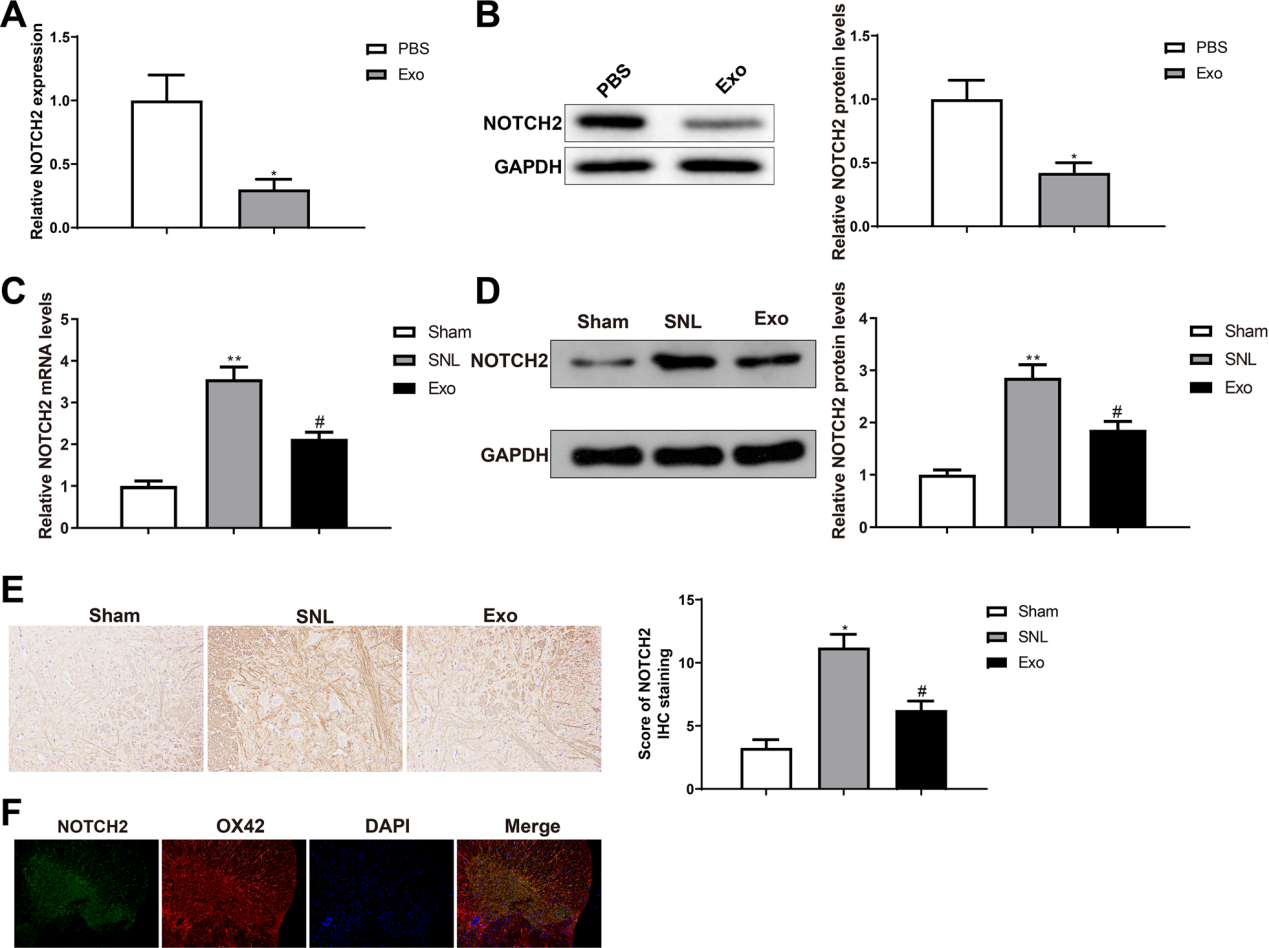
BMSC-exosomes downregulate microglial NOTCH2 expression. RT-qPCR (**A**) and western blots (**B**) were used to detect NOTCH2 expression in microglial cells after BMSC-exosome treatment; RT-qPCR (**C**), western blotting (**D**), and immunohistochemistry (**E**) were used to detect the expression of NOTCH2 in SDH; (**F**) Double immunofluorescence staining was used to localize NOTCH2. Data were shown as mean ± standard deviation. N = 6. ^*^P < 0.05, vs. the sham group; ^#^*P* < 0.05, vs. the SNL group. *BMSC* bone marrow mesenchymal stem cell, *SDH* spinal dorsal horn, *SNL* spinal nerve ligation

To determine the involvement of NOTCH2 in the therapeutic effects of BMSC-exosomes, we first analyzed the expression of NOTCH2 in microglial cells. RT-qPCR and western blotting showed that NOTCH2 expression was downregulated in microglial cells of the BMSC-Exo group versus the PBS group (Fig. 4A, B, *P < 0.05), suggesting the involvement of microglial NOTCH2 with mechanical allodynia. The mRNA and protein expression of NOTCH2 in rat SDH was increased in the SNL group compared with that in the sham group (*P < 0.05); NOTCH2 expression was suppressed in the Exo group than in the SNL group (^*#*^*P* < 0.01) (Fig. 4C, D). The results of IHC for NOTCH2 in rat SDH were consistent with those of RT-qPCR and western blotting for NOTCH2 (Fig. 4E). These results revealed that intrathecal injection of BMSC-exosomes blocked NOTCH2 expression in rat SDH. Furthermore, we used immunofluorescence to localize NOTCH2 in rat SDH. NOTCH2 was coexpressed with the microglial marker OX42 (Fig. 4F). The above results indicated that BMSC-exosomes suppressed the expression of NOTCH2 in microglial cells to mitigate mechanical allodynia in rats.

### BMSC-exosomes block mechanical allodynia in rats by inhibiting NOTCH2 expression

**Fig. 5:**
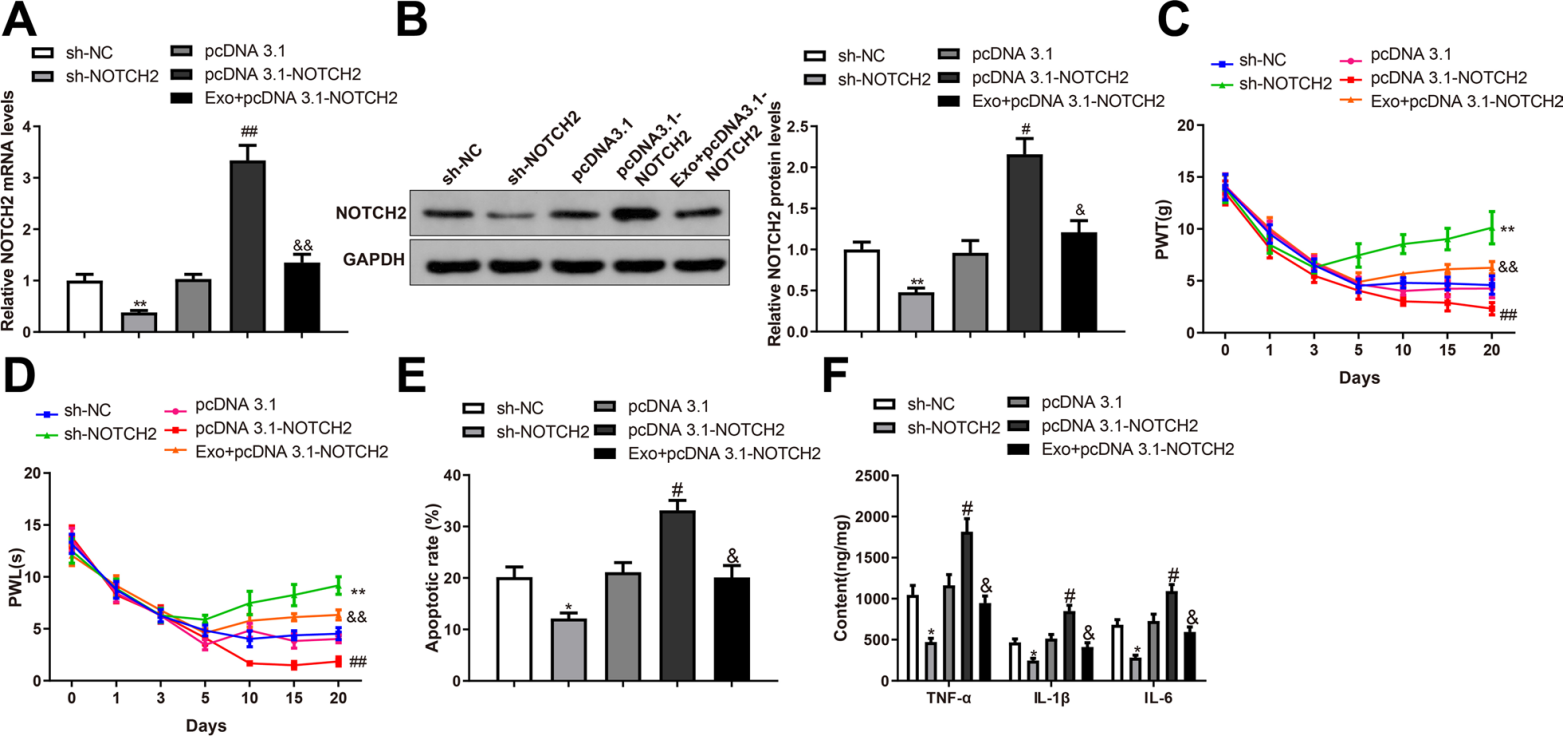
BMSC-exosomes suppress mechanical allodynia in rats by inhibiting the expression of NOTCH2. RT-qPCR (**A**) and western blotting (**B**) were used to detect the expression of NOTCH2 in rat SDH; Hind paw PWT (**C**) and PWL (**D**) of SNL rats were measured on days 0, 1, 3, 5, 10, 15, and 20 after surgery; (**E**) TUNEL staining revealed the apoptosis in SDH; (**F**) ELISA detected the expression levels of TNF-α, IL-1β, and IL-6 in the SDH. Data were shown as mean ± standard deviation. N = 6. ^*^P < 0.05, vs. the sh-NC group; ^#^*P* < 0.05, vs. the pcDNA3.1 group; ^&^*P* < 0.05, vs. the pcDNA3.1-NOTCH2 group. *BMSC* bone marrow mesenchymal stem cell, *SNL* spinal nerve ligation, *SDH* spinal dorsal horn, *PWT* paw withdrawal threshold, *PWL* paw withdrawal latency

To further investigate whether BMSC-exosomes acted on mechanical allodynia by regulating NOTCH2, we intrathecally injected pcDNA3.1-NOTCH2, pcDNA3.1, sh-NOTCH2, sh-NC, or BMSC-exosomes + pcDNA3.1-NOTCH2 into SNL rats. The mRNA and protein expression of NOTCH2 was downregulated in the sh-NOTCH2 group (*P < 0.05 vs. sh-NC group) and upregulated in the pcDNA3.1-NOTCH2 group (^*#*^*P* < 0.01 vs. pcDNA3.1 group); the levels of NOTCH2 mRNA and protein were reduced in the Exo + pcDNA3.1-NOTCH2 group compared with the pcDNA3.1-NOTCH2 group (^*&*^*P* < 0.01) (Fig. 5A, B). PWT and PWL of SNL rats were increased in the sh-NOTCH2 group (*P < 0.05 vs. the sh-NC group) and decreased in the pcDNA3.1-NOTCH2 group (^*#*^*P* < 0.01 vs. the pcDNA3.1 group); rats of the Exo + pcDNA3.1-NOTCH2 group showed increased PWT and PWL than in the pcDNA3.1-NOTCH2 group (^*&*^*P* < 0.01) (Fig. 5C, D). TUNEL staining demonstrated that the apoptotic rate in rat SDH was inhibited in the sh-NOTCH2 group (*P < 0.05 vs. sh-NC group) but promoted in the pcDNA3.1-NOTCH2 group (^*#*^*P* < 0.01 vs. pcDNA3.1 group); the apoptotic rate in rat SDH was ameliorated in the Exo + pcDNA3.1-NOTCH2 group compared with that in the pcDNA3.1-NOTCH2 group (^*&*^*P* < 0.01) (Fig. 5E). ELISA showed that the levels of TNF-α, IL-1β, and IL-6 in rat SDH were reduced in the sh-NOTCH2 group (*P < 0.05 vs. sh-NC group) and increased in the pcDNA3.1-NOTCH2 group (^*#*^*P* < 0.01 vs. pcDNA3.1 group); the expression of these inflammatory cytokines was suppressed in the Exo + pcDNA3.1-NOTCH2 group compared with that in the pcDNA3.1-NOTCH2 group (^*&*^*P* < 0.01) (Fig. 5F, P < 0.05). Taken together, BMSC-exosomes ameliorated mechanical allodynia by inhibiting the expression of NOTCH2.

### Exosomal miR-150-5p targets NOTCH2 in microglial cells

**Fig. 6:**
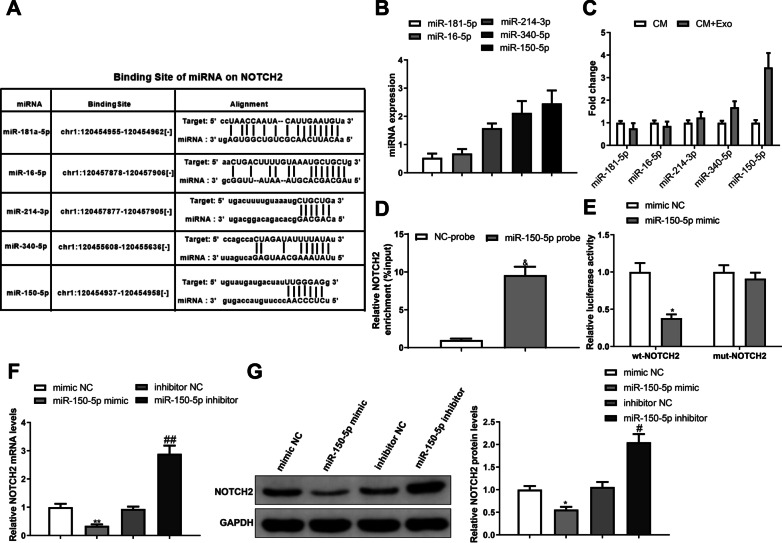
Exosomal miR-150-5p targets NOTCH2 in microglial cells. **A** StarBase predicted the miRNA-binding sites on NOTCH2; **B** RT-qPCR detected the expression of NOTCH2-binding miRNAs in BMSC-exosomes; **C** RT-qPCR detected the expression of the NOTCH2-binding miRNAs in microglial cells that were incubated with BMSC-exosomes or not; RNA pull down assay **D** and dual-luciferase reporter assay **E** verified the targeting relationship between miR-150-5p and NOTCH2; RT-qPCR **F** and western blotting **G** were used to detect the expression of NOTCH2 in microglial cells overexpressed or underexpressed miR-150-5p. Data were shown as mean ± standard deviation. Cellular experiments were repeated thrice. ^*^P < 0.05, vs. the mimic NC or CM group; ^#^*P* < 0.05, vs. the inhibitor NC group; ^&^*P* < 0.05, vs the NC-probe group. *BMSC* bone marrow mesenchymal stem cell

To further disclose the mechanisms operating by BMSC-exosomes alleviating mechanical allodynia in relation to NOTCH2, miRNAs that can interact with NOTCH2 were predicted using starBase database (http://starbase.sysu.edu.cn/), and the expression of these miRNAs was detected by RT-qPCR, which demonstrated that miR-150-5p had the highest expression in BMSC-exosomes (Fig. 6A, B). Additionally, BMSC-exosomes were cultured with microglial cells and the results suggested that the expression of miR-150-5p was increased in microglial cells (Fig. 6C, *P < 0.05). Therefore, we considered that BMSC-exosomes may regulate microglial NOTCH2 by delivering miR-150-5p. RNA pull down demonstrated that NOTCH2 could be captured by biotinylated miR-150-5p probes (Fig. 6D, ^&^*P* < 0.01). Dual-luciferase report assay (Fig. 6E, P < 0.01) showed that luciferase activity in microglial cells inserted with wt-NOTCH2 was reduced after transfection with miR-150-5p mimic, while luciferase activity of cells inserted with mut-NOTCH2 was unchanged after transfection with miR-150-5p mimic, indicating that miR-150-5p targeted NOTCH2 in microglial cells. Furthermore, the mRNA and protein expression of NOTCH2 was downregulated in microglial cells overexpressing miR-150-5p (*P < 0.05) and upregulated in microglial cells with low miR-150-5p expression (^*#*^*P* < 0.01) (Fig. 6F, G). From the above, miR-150-5p released from BMSC-exosomes can target NOTCH2 in microglial cells.

### Inhibition of miR-150-5p in BMSC-exosomes offsets the therapeutic effects of BMSC-exosomes in mechanical allodynia of rats

**Fig. 7:**
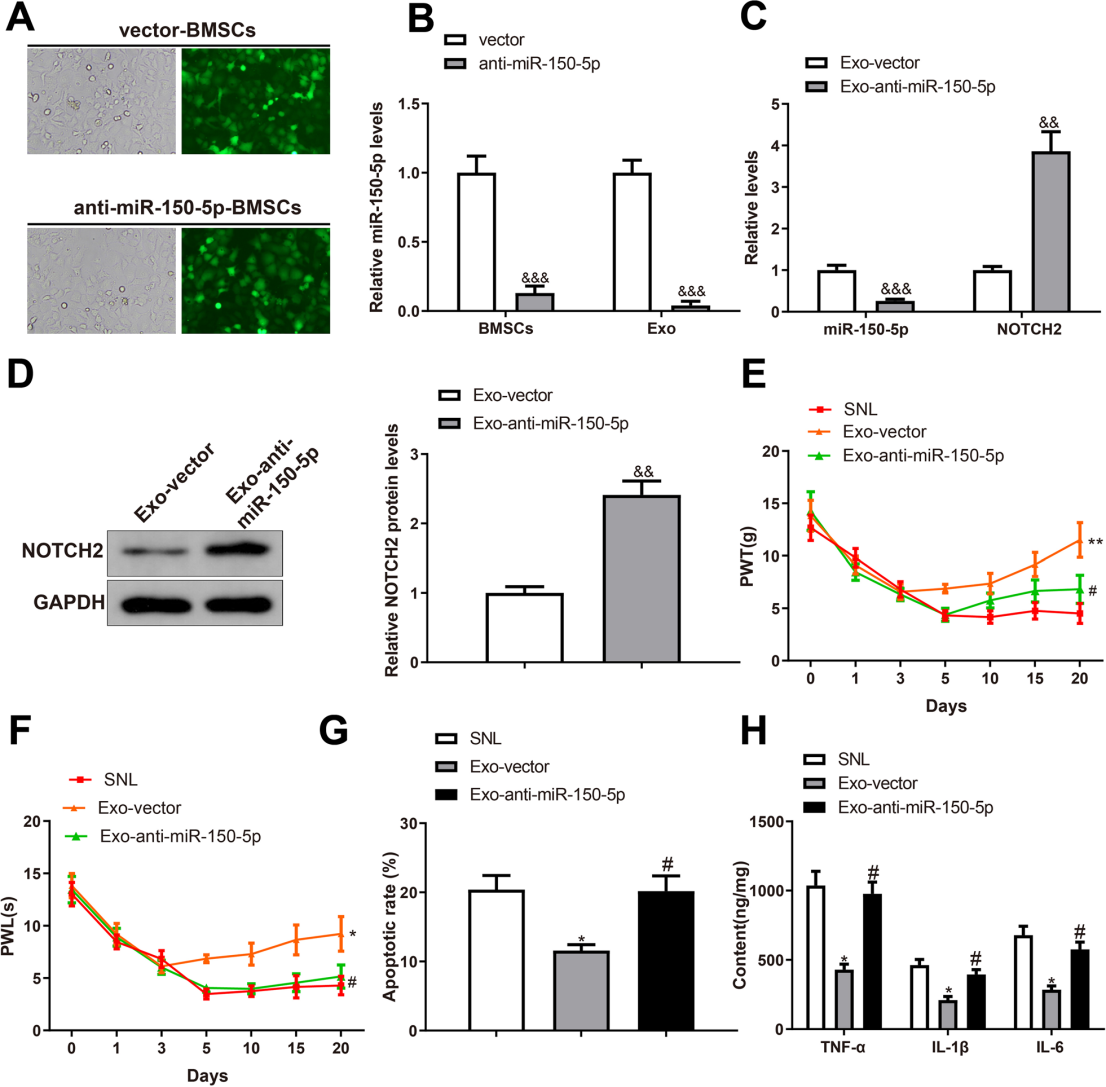
Inhibition of miR-150-5p in BMSC-exosomes offsets the therapeutic effects of BMSC-exosomes on mechanical allodynia. **A** The transfection efficiency was observed under a fluorescence microscope; **B** RT-qPCR was used to detect the expression of miR-150-5p in BMSCs and BMSC-exosomes. RT-qPCR **C** and western blotting **D** were used to detect the expression of miR-150-5p and NOTCH2 in the SDH of SNL rats; Hind paw PWT **E** and PWL **F** of SNL rats were measured on days 0, 1, 3, 5, 10, 15, and 20 after surgery; **G** TUNEL staining revealed the apoptosis in the SDH; **H** ELISA detected the expression levels of TNF-α, IL-1β and IL-6 in the SDH. Data were shown as mean ± standard deviation. N = 6. ^&^*P* < 0.05, vs. the vector or Exo-vector group. *BMSC* bone marrow mesenchymal stem cell, *SNL* spinal nerve ligation, *SDH* spinal dorsal horn, *PWT* paw withdrawal threshold, *PWL* paw withdrawal latency

BMSCs were infected with LV-anti-miR-150-5p or vector, and almost all cells were transfected with LV-anti-miR-150-5p under a fluorescence microscope (Fig. 7A). Exosomes were extracted from the infected BMSCs and miR-150-5p expression was detected by RT-qPCR. miR-150-5p was downregulated in both BMSCs and exosomes after transfection with LV-anti-miR-150-5p (Fig. 7B, ^&^*P* < 0.001). SNL rats were intrathecally injected with exosomes extracted from the infected BMSCs, and the expression of miR-150-5p and NOTCH2 in rat SDH was revealed by RT-qPCR and western blotting. miR-150-5p was downregulated while NOTCH2 was upregulated in the Exo-anti-miR-150-5p group compared with those in the Exo-vector group (Fig. 7C, D, ^&^*P* < 0.05).

Behavioral tests showed that PWL and PWT were decreased in the Exo-anti-miR-150-5p group compared with those in the Exo-vector group (Fig. 7E, F, ^&^*P* < 0.05), indicating exacerbated mechanical allodynia and thermal hyperalgesia of SNL rats after miR-150-5p inhibition in BMSC-exosomes. Moreover, the apoptotic rate and levels of TNF-α, IL-1β, and IL-6 were increased in the SDH of the Exo-anti-miR-150-5p group compared with those in the Exo-vector group (Fig. 7G, H, ^&^*P* < 0.05). Taken together, inhibition of miR-150-5p in BMSC-exosomes offsets the therapeutic effects of BMSC-exosomes on mechanical allodynia and SDH injury.

## Discussion

Neuropathic pain is a complex and multifactorial condition that is prevalent in patients with spinal cord injury. Although there are various pharmacological and non-pharmacological treatments available to patients with neuropathic pain, none of these medications is one size fits all, and some of them may have harmful or uncomfortable side-effects (Hatch et al. 2018). Therefore, more effective and personalized approaches are needed to reduce the healthy burden of patients with neuropathic pain. This study elucidates the therapeutic effects of BMSC-exosomes in the rat model with spinal injury and reveals an exosome-mediated molecular axis in microglial cells for mechanical allodynia.

First, we found that BMSC-exosomes ameliorated mechanical allodynia and thermal hyperalgesia in SNL rats, and reduced the apoptosis and inflammation in rat SDH. A previous study has reported that stem cells derived from bone marrow and umbilical cord can reduce allodynia and hyperalgesia in neuropathic pain after spinal cord injury (Yousefifard et al. 2016). Several studies have also investigated the mechanisms behind the analgesic effects of BMSCs. TSG-6 secreted by BMSCs alleviated neuropathic pain and neuroinflammation by inhibiting the TLR2/MyD88/NF-κB pathway in spinal microglial cells (Yang et al. 2020). IL-1β-pretreated BMSCs suppressed microglia activation and neuropathic pain by inhibiting the spinal expression of CCL7 (Li et al. 2017). Collected evidence suggests that spinal microglial cells function as a target for BMSCs in the treatment of neuropathic pain.

After peripheral nerve injury, microglial cells are activated by molecular mediators such as neuregulin-1, chemokine (C–C motif) ligand 2, and fractalkine; activated microglial cells in turn release IL-6, IL-1β, and TNF-α to cause painful symptoms (Zhao et al. 2017). To determine the involvement of microglial cells in the therapeutic effects of BMSC-exosomes, we intrathecally injected ATP-stimulated microglial cells that were pretreated with PBS or BMSC-exosomes into uninjured rats. Pretreatment of BMSC-exosomes attenuated neuropathic pain that was induced by activated microglial cells, and involved the activation of microglial cells. Based on these findings, we speculated that BMSC-exosomes acted on neuropathic pain by mediating the molecular signaling in microglia.

Transcriptome analyses have shown that a set of genes have altered expression in activated SDH microglia, and many of them are highly microglia selective such as interferon regulatory factor 8 (IRF8) which further regulates cell surface receptors and diffusible factors in activated microglia (Inoue and Tsuda 2018). The members of the NOTCH pathway have undeniable relevance with microglial activation (Guo et al. 2021). Reportedly, microglia activation and inflammation in spinal cord in response to diabetic neuropathic pain was dependent on NOTCH1 receptor (Yang et al. 2017). The presence of NOTCH1 irritated mechanical allodynia and thermal hyperalgesia threshold and induced TNF-α level in dorsal root ganglions from neuropathic rats (Chen et al. 2017). NOTCH intracellular domain (NICD) was upregulated in SDH after cystitis, and NOTCH inhibitor treatment attenuated mechanical allodynia and microglial activation (Chen et al. 2021).

In this study, we found that NOTCH2 was abundantly expressed in SDH microglial cells. Inhibition of SDH NOTCH2 ameliorated the symptoms of neuropathic pain in SNL rats and reduced inflammation and apoptosis in the SDH. In contrast, overexpression of NOTCH2 aggravated neuropathic pain and neuroinflammation in SNL rats, while BMSC-exosomes could block neuropathic pain promoted by NOTCH2 overexpression. Therefore, BMSC-exosomes exert the analgesic effects by inhibiting the expression of NOTCH2 in SDH microglia.

BMSC-derived conditioned medium has demonstrated to relieve hypoxia/reoxygenation-induced cardiomyocyte apoptosis and oxidative stress by inactivating the NOTCH2 signaling (Li et al. 2019). Human placenta-derived MSCs decreased peribronchial inflammatory cell infiltration by decreasing NOTCH-1/2 and jagged-1 levels (Li et al. 2018). However, the exact BMSC-derived molecule that inhibits the expression of NOTCH2 is not known. Considering exosomes as important carriers of miRNAs, we searched for NOTCH2-binding miRNAs that were expressed in BMSC-exosomes. Among those NOTCH2-binding miRNAs, miR-150-5p showed the highest expression in BMSC-exosomes. Moreover, miR-150-5p was highly expressed in microglial cells that were preincubated with BMSC-exosomes. Experiments further validated the binding of miR-150-5p to NOTCH2 in microglial cells.

miR-150-5p is a multifunctional transcript, but its function in neuropathic pain remains undiscovered. A number of studies have supported the tumor suppressor role of miR-150-5p in cancers (Chen et al. 2018a, b; Koshizuka et al. 2018; Lu et al. 2017). MSC-derived exosomal miR-150-5p suppressed migration and invasion of fibroblast-like synoviocyte and angiogenesis in rheumatoid arthritis (Chen et al. 2018a, b). In this study, we found that miR-150-5p-underexpressed BMSC-exosomes promoted the expression of NOTCH2 in the SDH of SNL rats. Inhibition of miR-150-5p exacerbated mechanical allodynia and thermal hyperalgesia in SNL rats, and promoted inflammation and apoptosis in the SDH.

## Conclusion

In summary, BMSC-derived exosomal miR-150-5p suppresses neuropathic pain by targeting NOTCH2 in SDH microglial cells. This study reveals an exosome-mediated molecular mechanism for neuropathic pain and gives novel insights into the feasibility of exosome-based cell-free medicine for neuropathic pain.

## Data Availability

The datasets used or analyzed during the current study are available from the corresponding author on reasonable request.
